# Recent advances in quantum dots-based biosensors for antibiotics detection

**DOI:** 10.1016/j.jpha.2021.08.002

**Published:** 2021-08-04

**Authors:** Rui Ding, Yue Chen, Qiusu Wang, Zhengzhang Wu, Xing Zhang, Bingzhi Li, Lei Lin

**Affiliations:** a School of Food Science and Pharmaceutical Engineering, Nanjing Normal University, Nanjing, 210023, China; b School of Nursing, Nanjing Medical University, Nanjing, 211166, China; c School of Environment, Nanjing Normal University, Nanjing, 210023, China; d Jiangsu Conat Biological Products Co., Ltd., Taixing, Jiangsu, 225400, China

**Keywords:** Biosensor, Antibiotic, Quantum dot, Molecularly imprinted polymer, Aptamer, Immunosensor

## Abstract

Antibiotics are a category of chemical compounds used to treat bacterial infections and are widely applied in cultivation, animal husbandry, aquaculture, and pharmacy. Currently, residual antibiotics and their metabolites pose a potential risk of allergic reactions, bacterial resistance, and increased cancer incidence. Residual antibiotics and the resulting bacterial antibiotic resistance have been recognized as a global challenge that has attracted increasing attention. Therefore, monitoring antibiotics is a critical way to limit the ecological risks from antibiotic pollution. Accordingly, it is desirable to devise new analytical platforms to achieve efficient antibiotic detection with excellent sensitivity and specificity. Quantum dots (QDs) are regarded as an ideal material for use in the development of antibiotic detection biosensors. In this review, we characterize different types of QDs, such as silicon, chalcogenide, carbon, and other doped QDs, and summarize the trends in QD-based antibiotic detection. QD-based sensing applications are classified according to their recognition strategies, including molecularly imprinted polymers (MIPs), aptamers, and immunosensors. We discuss the advantages of QD-derived antibiotic sensors, including low cost, good sensitivity, excellent stability, and fast response, and illustrate the current challenges in this field.

## Highlights

• The strategies of applying quantum dots (QDs) in antibiotic detection are described.
• The QD-based sensors of antibiotics are first summarized.
• Sensors are classified according to antibiotic-recognition strategy.
• QD antibiotic detection abilities and advantages are described.

## Graphical abstract

**Figure:**
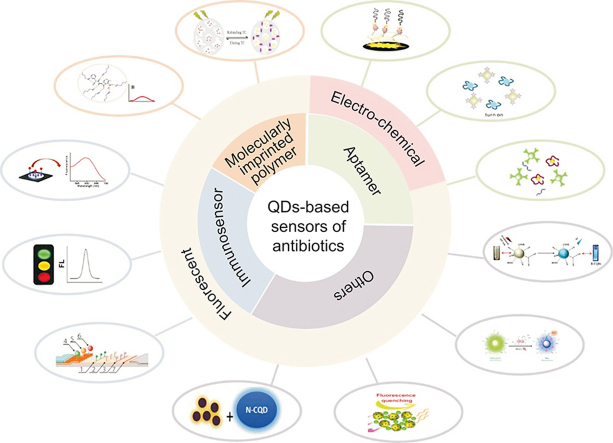

## 1 Introduction

Antibiotics, considered as “wonder drugs,” are chemical compounds used to treat bacterial infections and are widely applied in clinical medicine, cultivation, animal husbandry, and aquaculture [[1], [2], [3]]. Antibiotics can be classified, according to their structure, into tetracyclines, macrolides, aminoglycosides, streptogramins, β-lactam antibiotics, quinolones, and peptides [1,4]. A brief list of commonly used antibiotics was selected and is summarized in Table 1 [[5], [6], [7], [8], [9], [10], [11], [12], [13], [14], [15], [16], [17], [18], [19], [20], [21]]. A variety of antibiotics have been shown to have growth-promoting effects at a sub-therapeutic dose level based on the alteration of intestinal flora, and the effect was verified to be strongly associated with metabolism and immunity in the body [1,22]. Antibiotics can suppress cell wall synthesis, curtail bacterial DNA synthesis, inhibit protein synthesis, block nascent RNA extension, and restrict the transcription cycle, thus leading to the elimination of microbial communities [3]. Therefore, antibiotics enhance our ability to combat bacterial infections, greatly reducing the impact of such infections on human mortality and maintaining both human and animal health.

**Table 1:** Representative classes of antibiotic contaminants.

Type of antibiotics	Representative members	Consumers	Side-effects	Refs.
Tetracyclines	Tetracycline, oxytetracycline, chlortetracycline, rolitetracycline, doxycycline	Human, veterinary, agriculture	Ecological risks, human health damage, endocrine disorder of aquatic species	[5,6]
Macrolide	Erythromycin, clarithromycin, roxithromycin, azithromycin, josamycin	Human, veterinary	Sharply reduced biological activity, decreased shelf-life, human health damage	[[7], [8], [9]]
Aminoglycosides	Streptomycin, neomycin, gentamicin, tobramycin	Human, veterinary	Ototoxicity, nephrotoxicity	[[10], [11], [12]]
β-lactam	Penicillins, cephalosporins, carbapenems, monobactams	Human, veterinary	Rashes, fever	[[13], [14], [15], [16]]
Streptogramins	Mikamycin, ostreogrycin	Human	Myalgia and arthralgia	[17,18]
Peptides	Tyrocidine, cyclomarin, reginamide	Human, veterinary	Stability problem, poor cell membrane permeability	[19,20]
Quinolones	Ciprofloxacin, levofloxacin, moxifloxacin, norfloxacin	Human, veterinary	Tendinitis and tendon rupture, severe hepatic toxicity	[21]

Antibiotics used in humans, plants, and animals are only partially metabolized and can therefore be introduced in the environment through various excretion pathways in complete or resolved forms, such as wastewater discharge, agricultural land runoff, or human excreta [1,2,23]. For example, it was found that the concentrations of erythromycin, sulfamethoxazole, and trimethoprim were 0.10–16.6 ng/L in the offshore waters of the Yellow Sea and the Bohai Sea [23]. These residual antibiotics or their metabolites pose a potential risk of causing antibacterial resistance in sensitive aquatic organisms and can be absorbed by humans through the food chain, leading to allergic reactions, bacterial resistance, and increased cancer incidence [1]. Residual antibiotics in the environment can cause negative effects, including damaging the ecological health of water and causing abnormal growth of water organisms and imbalance of ecosystems [24]. Studies have revealed that the intake of antibiotics via drinking water increases the risk of severe human diseases, including cancers [24,25]. The gut microbes of the human body can be disturbed by antibiotic residues in drinking water, and long-term exposure to antibiotics can lead to changes in the intestinal microflora structure, thus increasing the opportunities for the appearance of antibiotic-resistant harmful bacteria in humans [25]. Moreover, owing to improvements in drug stability of antibiotics, many antibiotics can persist in the environment for a long time [26]. Lyu et al. [27] summarized antibiotic detection rates in soil and water in China, showing that the detection rates of antibiotics in soil, surface water, and coastal waters were 100%, 98.0%, and 96.4%, respectively. Li et al. [28] found that antibiotics pose high ecological risks to aquatic organisms (algae and plants) in surface water. It is well established that environmental antibiotics cause pollution owing to concealed hostile effects on human health and ecological systems [3]. Recent reports suggest that the death of 700,000 people globally could be attributed to antimicrobial resistance, such as in the “superbugs” methicillin-resistant *Staphylococcus aureus* (MRSA) [29]. Therefore, it is necessary for effective treatment to rapidly discriminate MRSA because such bacteria are difficult to treat with the existing medicines, even in well-equipped hospitals [30]. In addition, antimicrobial resistance causes more than two million infections owing to antibiotic treatments in the US alone, which brings an extra cost of 20 billion dollars to the US health system annually [30]. Residual antibiotics and antibiotics resistance have become a global challenge and focus of attention [31]. To overcome these challenges, the straightforward monitoring of antibiotics is highly desirable to reduce potential environmental contamination and ecological health risks from antibiotic pollution [32]. However, current methods for the detection of antibiotics, such as chromatography-mass spectrometry, usually have relatively tedious procedures and high costs, which restricts their wide applications, particularly in on-site monitoring [33]. Therefore, to protect human and environmental health, it is important to develop new analytical platforms for efficient, sensitive, and specific detection of antibiotics. Such analytical techniques are also valuable for the development of antibiotic analysis devices.

In recent years, quantum dots (QDs) have been recognized as an ideal material for the development of biosensors for antibiotic detection. QDs are a type of novel fluorescent nanomaterial consisting of inorganic nuclei with organic molecules in the nanoscale range of 1–10 nm applied to the surface of the nucleus [[34], [35], [36]]. These materials usually consist of carbon, silicon, cadmium selenide, cadmium sulfide, or indium arsenide and emit fluorescence when excited by a light source [37]. QDs possess unique chemical properties and excellent optical properties, including extended fluorescence lifetime, adjustable particle sizes, superior signal brightness, emission of multiple fluorescence colors, confined emission spectra, and broad excitation spectra. They are widely used in pharmaceuticals, biosensors, biology, real-time tracking, multi-color labeling, and imaging [36,[38], [39], [40]]. For example, carbon-based dots (CDs) can be applied in biomedicine, bioimaging, photocatalysis, and detection of various analytes [41]. Additionally, the abundant functional groups on QDs have made it easy to form hybrid nanomaterials with advanced analytical performance. By hybridizing QDs with nanotubes, molecularly imprinted polymers (MIPs), noble metal nanoparticles, and fluorescent nanoclusters, the as-prepared nanomaterial can integrate the excellent optical properties of QDs into functionalized sensing systems, realizing the sensitive and selective detection of multiple antibiotics, biomarkers, and metal ions. For example, an rAu-Pt-nanoparticles/graphene quantum dots/glassy carbon electrode (rAu-PtNPs/GQDs/GCE) developed by Yola and Atar [42] can achieve the multiplexed detection of tryptophan, ascorbic acid, uric acid, and dopamine with high sensitivity and specificity. Researchers have developed carbon nitride nanotubes@GQDs (C_3_N_4_ NTs@GQDs) for chlorpyrifos detection in wastewater samples [43], and prepared GQDs/functionalized multi-walled carbon nanotubes (f-MWCNTs) as a new molecularly imprinting biosensor for interleukin-6 protein detection [44]. Other researchers have provided a novel imprinted biosensor approach based on boron nitride QDs (BNQDs) to detect cardiac troponin-I in plasma samples [45] and produced a new electrochemical sensor to determine citrinin (CIT) in chicken egg samples [46]. Hong et al. [47] built a novel ratiometric fluorescence nanosensor on gold nanoclusters (AuNCs) and GQDs that was applied to detect glucose in humans and also has great potential for the determination of other physiological substances and their corresponding oxidases. Mehrzad-Samarin et al. [48] constructed a GQD-embedded silica molecularly imprinted polymer (SMIP) as an optical nanosensor for metronidazole (MNZ) in the plasma matrix. Song et al. [49] created a fluorescence nanosensor using carboxymethyl chitosan-modified CdTe QDs (CMCS-QDs) for lysozyme detection in human serum samples with ultrasensitivity, excellent selectivity, and convenience. Mou et al. [50] developed a novel fluorescent nanosensor to detect Zn^2+^ in water samples on Cu–In–S QDs, which could also be used in the fields of biomedicine and the environment. Therefore, QDs are highly promising nanomaterials in engineering as sensing components for molecular recognition and target detection.

Nevertheless, though various studies regarding antibiotic analysis based on QDs have been published, there are only very few reports summarizing their applications. In the current review, we first introduce the research trends in the preparation of QDs and then discuss their applications in the determination of antibiotics. We believe that the study of QD-based biosensors will accelerate the emergence of conventional analysis tools that aid the convenient monitoring of antibiotics.

## 2 Overview of QDs utilized for antibiotics sensing

**Fig. 1:**
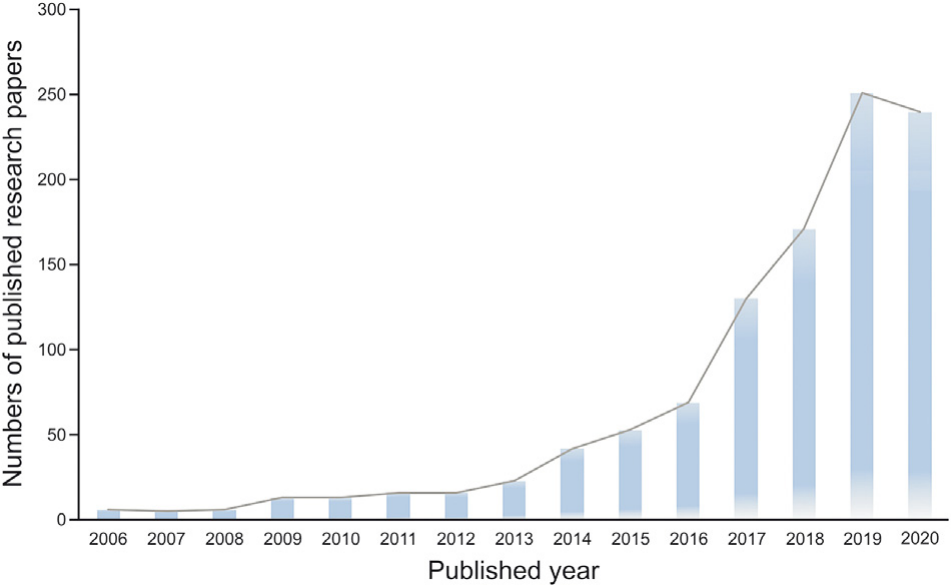
Collected statistics of published research papers on QD-based sensors for the determination of antibiotics. (Adapted from ISI Web of Science, retrieve the article date from 2006 to 4 March, 2021).

The increasing number of published reports illustrated in Fig. 1 indicates that QDs have been flourishing as promising tools in the development of biosensors targeting antibiotics. The selected methods for the detection of common antibiotics based on various QDs are summarized in Table 2 [33,[51], [52], [53], [54], [55], [56], [57], [58], [59], [60]], where the comments, limit of detection (LOD), and matrices are listed. In addition, there are many other reports on the detection of antibiotics based on QDs. Chen et al. [61] produced a useful magnetic-QDs (MNPs-SiO_2_-QD) material based on magnetic nanoparticles (MNPs) and QDs for qualitative and quantitative detection of four antibiotics with a high percent recovery. Meng et al. [62] built a novel method that could measure five quinolone antibiotic residues in foods using cadmium telluride (CdTe) QDs as a fluorescent background material. Combining GQDs and Eu^3+^, Li et al. [63] established a system to detect tetracyclines (TCs), and this system showed remarkable potential for the detection of TCs in environmental and biological samples. Zhang et al. [51] synthesized a novel fluorescence probe using the hybrid QD/mesoporous silica/MIP (QD/MS/MIP) to detect tetracycline (TC) in serum samples. Using QDs as the fluorescent marker, Chen et al. [64] established a competitive fluorescence-linked immunosorbent assay (cFLISA) to detect enrofloxacin (ENR) in chicken muscle tissue, and cFLISA is suitable for screening veterinary drug residues. Several kinds of QDs have been developed and functionalized to detect antibiotics.

**Table 2:** Performance analysis of antibiotics detection based on different quantum dots.

Methods	Comments	Limit of detection	Matrices	Refs.
QDs/MS/MIP	Uncomplicated preparation process, great selectivity and stability, low cost, and celerity	15.0 ng/mL	Serum	[51]
Mn:ZnS/SiO_2_/QDs/MIP	Low toxicity, long decay time, short-lived autofluorescence, minimal light scattering interference, high sensitivity and selectivity, and good binding capacity	0.81 μg/L	Raw milk and milk powder	[33]
AgNPs/GQDs-N-S/AuNPs/GCE	Low cost, simple, excellent thermal stability, high sensitivity, and rapid response	0.0033 pg/mL	Serum	[52]
SSB/QDs	A wide linear range, low detection limits, simple, and sensitivity	0.03 ng/mL	Milk	[53]
CdSe/dsDNA/QDs	High sensitivity and specificity, high selectivity and recyclability, and simple	0.002 ng/mL	Milk	[54]
Fluorescent immunoassay/QDs-antibody	Speedy detection, straightforward operation, outstanding sensitivity and selectivity, and effective	Aqueous solution: 1 pg/mL; milk: 10 pg/mL	Milk	[55]
Multicolor/QDs-Ab	Simultaneous analysis of multiple target antibiotics, remarkable accuracy and sensitivity, visual detection, and high throughput analysis	0.005 ng/mL	Milk	[56]
“Traffic light” immunochromatographic/multicolor/QDs	A wide working range, high reproducibility, high sensitivity, and near complete analyte recovery	Ofloxacin: 0.3 ng/mL; chloramphenicol: 0.12 ng/mL; streptomycin: 0.2 ng/mL	Milk	[57]
N-CQDs/Co_3_O_4_/MWCNT/GCE	High stability, high mechanical strength, and simultaneous determination	FLU: 0.0169 μM; NF: 0.044 μM	Urine	[58]
CdS_x_Se_1−x_/QDs	Good water solubility, excellent fluorescence properties, and high sensitivity	0.89 μg/L	Milk	[59]
Smartphone-based/CdTe QDs	Simple, rapid, low-cost, and on-site	0.26 nM	Milk and water	[60]

QD: quantum dots; MS: mesoporous silica; MIP: molecularly imprinted polymer; GQDs: graphene QDs; GCE: glassy carbon electrode; SSB: single-stranded DNA binding protein; QD-Ab: QDs-antibody; CQD: carbon QD; MWCNT: multi-walled carbon nanotube; FLU: flutamide; NF: nitrofurantoin.

At present, QDs can be roughly divided into silicon QDs (Si QDs), chalcogenide QDs, CDs, and doped QDs based on their formation. To improve readers' understanding of their applications in the sensing of antibiotics, next we describe the fundamentals of these four types of QDs, including their structures, applications, advantages, and disadvantages.

Si QDs can be created via different synthetic means, including electrochemical etching, ultrasonic/microwave synthesis, gas-phase laser light pyrolysis, chemical/photochemical reduction, thermal recovery of silicon oxides, Zintl salt metathesis, and disintegration in a supercritical solvent [65]. Si QDs have potential applications in the biological field because silicon exhibits good biocompatibility, and silicon-based materials are typically abundant, cheap, and nontoxic [66,67]. Moreover, Si QDs exhibit excellent biodegradation properties, water solubility, and strong quantum effects, making them appliable in biological fields [66,68]. For example, Si QDs have been applied in the controlled release of antibiotics such as the hydrolysis of amoxicillin (AMX) from the surface of Si QDs [65]. However, Si QDs with oxide surface passivation are rarely applied in biological imaging because Si QDs typically show dipole-forbidden yellow-red emission, and their synthesis is quite challenging [68].

Chalcogenide QDs are the most important fluorescent probes in the VI group, which are prepared from metallic chalcogenide substances including cadmium-selenium (CdSe), CdTe, cadmium-sulfur (CdS), and zinc-sulfur (ZnS) and have generated considerable interest for their optical and electrochemical properties [59]. Chalcogenide QDs show high stability, wide bandgap, surpassing water solubility, and excellent fluorescence properties, which enable their wide applications in in vivo imaging, diagnosis, and in optoelectronic devices with a range of 1–20 nm [69]. Chalcogenide QDs have a narrow emission profile with a half-maximum emission band of 20 nm, which decreases spectral overlap and enables multiplexed detection with the discrimination of different-colored QDs [70]. In addition, CdTe QDs have the potential to be applied in cancer diagnosis and therapy because of their large two-photon absorption cross-section, photostability, and biocompatibility. It has also been reported that their large surface area can be conjugated with targeting ligands for the customized delivery of molecular cargos [69,71].

CDs are small carbon fluorescent nanoparticles in the nanometer range with diverse surface passivation through chemical modification or functionalization, usually including carbon QDs (CQDs) and GQDs with sizes less than 10 nm and less than 100 nm inside dimensions, respectively [72,73]. Accordingly, the structures of CQDs and GQDs are dissimilar; GQDs generally have a smaller size and higher crystallinity, such that the graphite in-plane lattice distance is 0.18–0.25 nm and its interlamination distance is 0.32–0.34 nm or larger for distinct diffraction planes [72]. In addition, CQDs are usually composed of amorphous to nanocrystalline cores, which are an admixture of predominant sp^2^ hybridized carbons and sp^3^ diamond-like carbons [73]. Owing to their various compositions and structural variabilities, CDs demonstrate excellent performance in terms of high chemical stability, low toxicity, good biocompatibility, high water solubility, and unique photoluminescence [73,74]. In addition, CDs can engender optical excitation as a result of π-plasmon absorption in the core carbon nanoparticles, rather than band-gap absorptions of the quantum confinement effect [35,75,76].

To improve the photoluminescence qualities of QDs, doping technology has gradually been applied to develop original QDs with excellent functions and exceptional properties. Numerous ingredients can be merged into the main lattices of primigenial QDs via the doping procedure. For example, some elements (B, N, and S) can be doped into CQDs and a great diversity of transition metals can be doped into chalcogenide QDs, such as Cu, Mn, Fe, and Cd [77]. B-doped CQDs (B-CQDs) are fabricated via suitable hydropyrolysis of boric acid and citric acid and comprise multilattice planes with a mean diameter of 2.3 nm. B-CQDs are characterized by a superior linear range, high sensitivity, and relative selectivity. They have been applied to the detection of AMX at a concentration of 1.43–429.12 μmol/L in aqueous solution with an LOD of 0.825 μmol/L. In addition, their fluorescence property was enacted on common metallic ions, amino acids, and saccharides [78]. Several complex matrices may reduce the accuracy of fluorescent probes because of their intense background fluorescence or scattered light. To overcome this shortcoming, Mn:ZnS (Mn-doped ZnS) QDs can be prepared by simple sediment progress or sol-gel polymerization and have successfully acted as a photocatalyst for antibiotics and been used to detect antibiotic residues [79]. Mn:ZnS QDs have been utilized for their weak light scattering interference, low toxicity, long decay time, and short life of autofluorescence. In addition, synthesized probes using Mn:ZnS QDs show the advantages of high selectivity, good binding capacity, and excellent sensitivity with an LOD of 0.81 μg/L for the detection of antibiotics [33].

## 3 Recent advances in sensing applications

### 3.1 Molecularly imprinted polymer

MIPs are a type of polymeric material based on the complexation of a template molecule and a functional monomer in a suitable solvent via either covalent or non-covalent bonds. MIPs have the ability to recognize and bind specific target molecules [33,51,80,81]. To synthesize MIPs, monomers are co-polymerized via interactions and cross-linking and arranged based on the target molecules as a template for forming a shell; in other words, a molecule is marked in the polymer from memory that can be recognized and bind the target selectively [[82], [83], [84]].

**Fig. 2:**
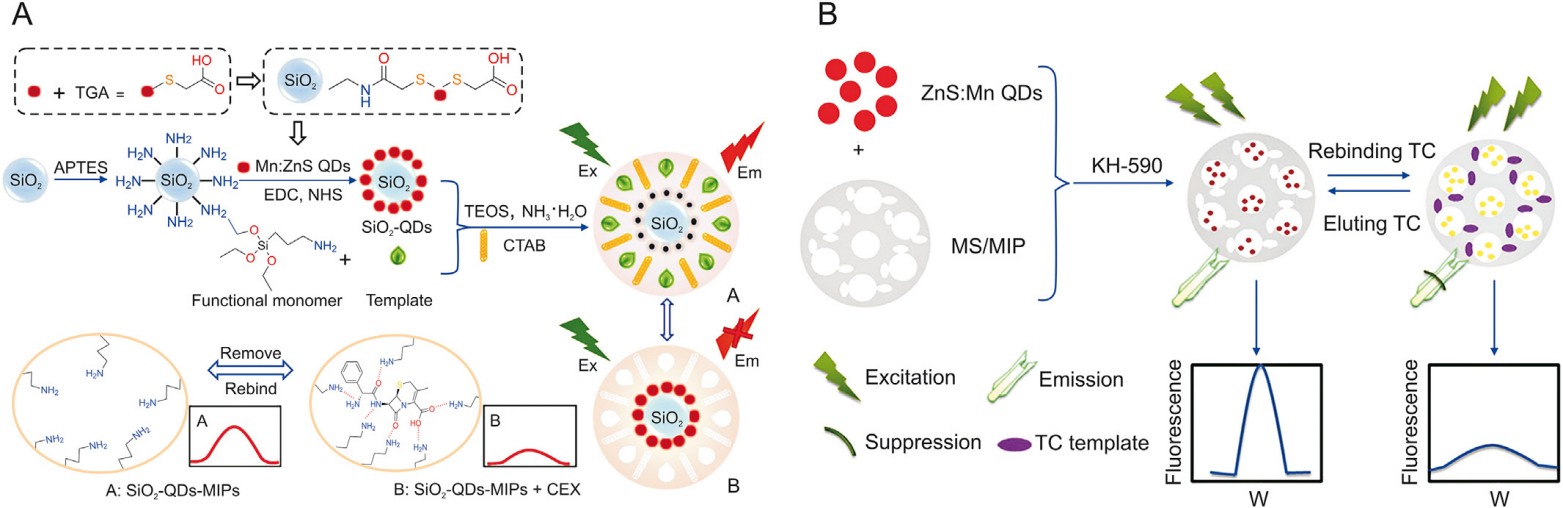
(A) Schematic diagram of the preparation of the SiO_2_-QD-MIP phosphorescence probe with mesoporous structure and the sensing mechanism for cephalexin (CEX). (B) Illustration of the preparation procedure of QD/MS/MIP fluorescence probe and the detection of tetracycline (TC). SiO_2_-QD-MIPs: SiO_2_-QD-molecularly imprinted polymers; QD/MS/MIP: QD/mesoporous silica/molecularly imprinted polymer. (Reprinted from Refs. [33,51] with permission).

Cephalexin (CEX) is a first-generation β-lactam antibiotic. Excessive retained CEX may pose health risks and increase the resistance of microorganisms to antibiotics. Thus, the residue limit of CEX is strictly controlled [13,15]. For example, the European Economic Community has defined the maximum limit value of CEX residue as 100 μg/L in milk. Chen et al. [33] developed a type of SiO_2_-QD-MIP phosphorescent probe using sol-gel polymerization, based on thioglycolic acid (TGA)-modified QDs as luminescent materials, 3-aminopropyl triethoxysilane as the functional monomer, CEX as the template, and tetraethoxysilane as the crosslinking agent (Fig. 2A) [33]. This probe was successfully utilized in the detection of CEX in powdered milk and raw milk samples with an LOD of 0.81 μg/L. Furthermore, SiO_2_-QD-MIPs, which combine the excellent selectivity of MIPs and phosphorescence probes, have greatly improved the response time by reducing the mass transfer resistance of the mesoporous structure. Nevertheless, this method cannot simultaneously monitor different target analytes in real samples.

TCs are highly oxidized type II polyketides that can restrict protein synthesis by preventing the attachment of ribosomal acceptors by aminoacyl-tRNA, showing activity in a wide range of atypical organisms and gram-negative and gram-positive bacteria [[85], [86], [87]]. Zhang et al. [51] achieved TC detection via a fluorescence probe using a hybrid QD/mesoporous silica/MIP (QD/MS/MIP) through the fluorescence quenching mechanism of photoinduced electron transfer (PET) (Fig. 2B) [51]. The template, TC, was combined with the monomer via covalent bonding. A hydrogen binding site was formed after the removal of the template, which was shown among the hole in the material and the target molecule. Moreover, the fluorescence quenching of the QDs emerged because a new compound can obtain the amount of energy of QDs and it was formed between the amino group of QD/MS/MIP and the hydroxyl group of TC during rebinding TC. The QD/MS/MIP fluorescence intensity was reduced in 10 min, and this composite material exhibited outstanding linearity from 50 to 1000 ng/mL, which is useful in fluorescence probe applications. The advantages of this designed fluorescence probe include enhanced binding ability of the specific target, simplicity, high stability, excellent selectivity, short analytical time, and low cost. The recovery was 90.2%–97.2% when spiking the targets in serum samples with relative standard deviations in the range of 2.2%–5.7%, and the LOD was 15.0 ng/mL.

### 3.2 Aptamers

Aptamers are short, single-stranded nucleic acid sequences that bind specific molecules with good affinity and selectivity. They are created by a conjunction technology of systematic transformation of ligands with exponential evolvement [[88], [89], [90]]. Owing to their advantages of high binding affinity, low immunogenicity, easy modification, long-term stability, and cost-effectiveness, aptamers have been extensively used in fields such as therapeutics, biomedicine, biosensing, gene therapy, cancer cell imaging, and targeted drug delivery [91].

**Fig. 3:**
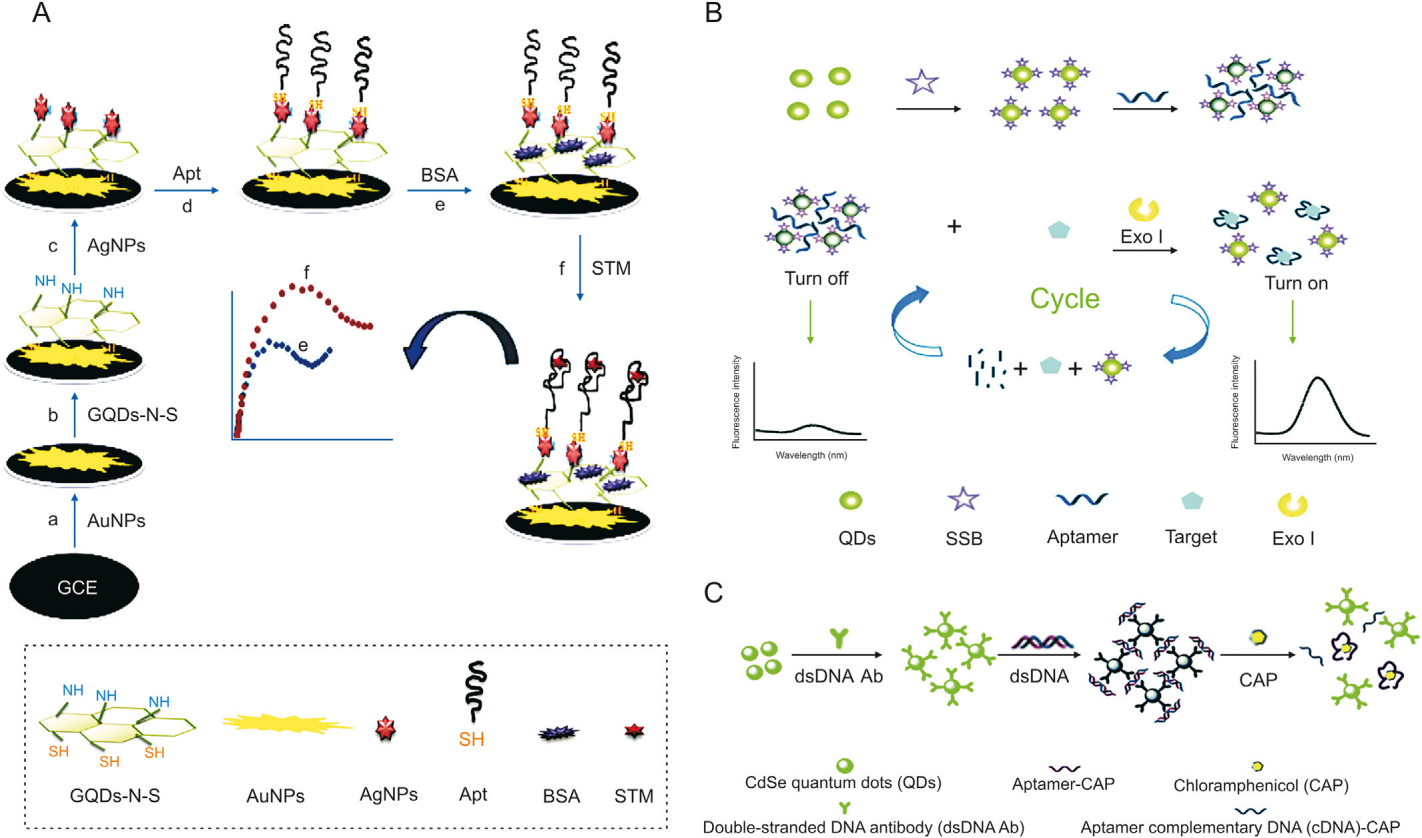
(A) Schematic diagram of the electrochemical aptasensor based on immobilization of the aptamer onto nanocomposite for streptomycin (STM) detection. (B) Schematic illustration of the composite of the fluorescent switch probe and the mechanism for the detection of STM using the fluorescent switch aptasensor. (C) Schematic representation of fluorescence assay for detecting chloramphenicol (CAP). (Reprinted from Refs. [[52], [53], [54]] with permission).

Streptomycin (STM) is an aminoglycoside widely used for the treatment of gram-negative pathogen infections [12]. Roushani et al. [52] designed an aptasensor using AgNPs/GQDs-N-S/AuNPs nanocomposite attached to the GCE surface for development of a novel and highly sensitive detection method for STM, and it functions in a low concentration at the level of pg/mL (Fig. 3A). AuNPs were first subsided on the GCE surface, followed by modification with GQDs-N-S via coupling between thiol and amine groups. Subsequently, GQDs-N-S/AuNPs/GCE was wrapped with silver nanoparticles, and the STM thiol-aptamer (SH-Apt) was combined with the resulting nanocomposite via Ag–S bond formation. This method holds significant benefits such as a simple preparation procedure, excellent sensitivity and stability, high speed of response, low cost, and good reproducibility and will be powerful for the detection of other targets.

The synthetic probe needs to adapt to the length of the complementary chain according to the DNA sequence of diverse aptamers, which is complex and time-consuming [53]. To address this issue, single-stranded DNA binding protein (SSB) is commonly applied because SSB can combine with the probe label on the aptamer, and it does not combine with the target captured by the aptamer [54]. For example, SSB can bind to single-stranded DNA (ssDNA) via specific binding without a coupling agent and tightly connect to dissociative ssDNA rather than double-stranded DNA (dsDNA). Using STM as a model, Wu et al. [53] designed an original fluorescent “signal-on” switch aptasensor using an SSB-labeled QD aptamer probe and exonuclease-supported target recycling for detecting antibiotics in homogeneous media (Fig. 3B). An aptamer is a bridging ligand that could blend with SSB, and the QDs distributed in the liquor are gathered by the aptamer, which causes a self-quenching phenomenon of the QD fluorescence. The aptamer preferentially binds the STM after mixing with the fluorescent probe and Exo I, which digests the target aptamer into mononucleotides and produces a strong fluorescence signal during the reaction cycle. This aptasensor could achieve the sensitive detection of STM with an LOD of 0.03 ng/mL and is suitable for a homogeneous sensing system.

Wang et al. [54] established a fluorescence assay using unique aptamer-dsDNA-QD probes to distinguish antibiotic residues (Fig. 3C). This sensor was constructed by combining dsDNA with CdSe QDs via covalent bonding. The probes contacted each other by forming dsDNA as a bridge, which led to fluorescence quenching. In a reaction system, after adding chloromycetin (CAP), the aptamer recognized CAP and exposed ssDNA via the dissociation of dsDNA, leading to the disassembly of the probe and enhancement of fluorescence. These probes were reused 10 times with recovery above 90% and applied for the detection of CAP with an LOD of 0.002 ng/mL.

### 3.3 Immunosensors

Immunosensors belong to a type of analytical biosensors that use antibodies (Abs) to recognize the antigens at the molecular level, which can be associated in highly specific chemical reactions with satisfactory physical transducers [92]. Owing to their unique selectivity, excellent sensitivity, flexible application, and ease of handling, immunosensors have been widely applied in fields such as food safety evaluation, environmental monitoring, and medical diagnosis. In particular, they have been successfully used in medical diagnostics for cancer, infections, autoimmune, and cardiovascular diseases, and allergies [39,[93], [94], [95]].

**Fig. 4:**
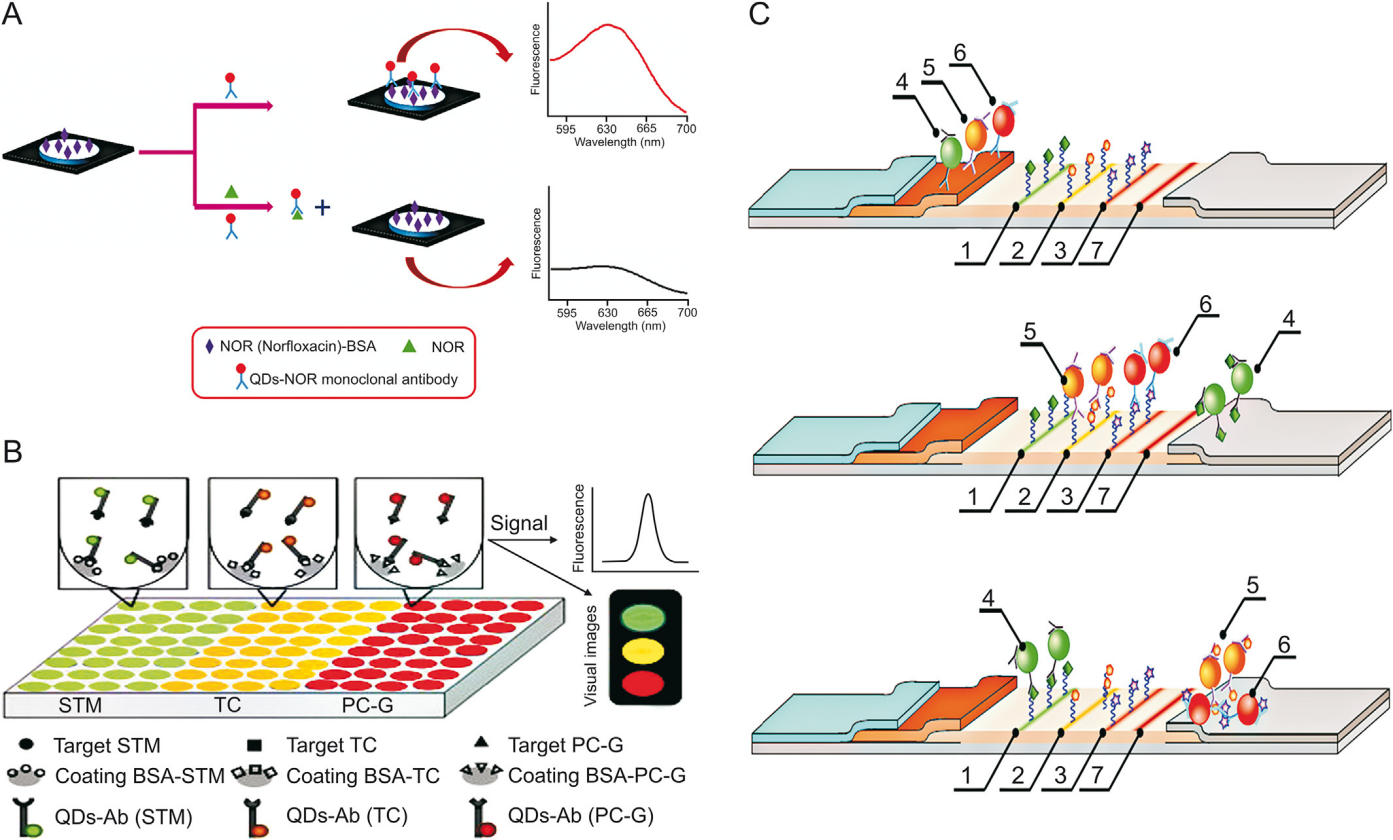
(A) Schematic illustration of the detection of norfloxacin (NOR) by paper-based fluorescent immunoassay. (B) Schematic diagram of the detection of three kinds of antibiotics based on conventional fluorescence immunoassay (cFIA) by QD-Ab. (C) Principle of the competitive detection of three antibiotics based on “traffic light” immunochromatographic test (1: test zone for STM; 2: test zone for CAP; 3: test zone for ofloxacin (OFL); 4: conjugate QD-mAb/STM; 5: conjugate QD-mAb/CAP; 6: conjugate QD-mAb/OFL; 7: control line). QD-Ab: QD-antibody probes; QD-mAb: QD-monoclonal antibodies. (Reprinted from Refs. [[55], [56], [57]] with permission).

Norfloxacin (NOR) is usually used for the treatment of veterinary diseases. However, excessive NOR remaining in animal food such as milk may affect mammalian cell duplication and decrease the efficiency of treating infections [21]. Zong et al. [55] developed a paper-based fluorescent immunoassay based on a QD-labeled NOR monoclonal antibody as a probe to detect NOR at the picogram level in milk (Fig. 4A). Pre-immobilized NOR-BSA on paper could capture the QD-labeled antibodies, generating fluorescence. The fluorescence intensity decreased with increased concentrations of NOR. In addition, because antibodies targeting different antibiotics can be labeled with different QDs possessing different emission wavelengths, this method has the potential of multiple antibiotics detection, simultaneously in diverse zones of the paper.

Song et al. [56] established a strategy to achieve the simultaneous analysis of multiple antibiotics in milk using one assay using the combination of a multicolor QD-based immunofluorescence measurement with a method of sequential analysis (Fig. 4B). The detection probes (QD-Ab) were made from Abs for STM, TC, and penicillin (PC-G) combined with QDs having distinct emission wavelengths (QD520 nm, QD565 nm, and QD610 nm). This allowed a fluorescent immunoassay with direct competition to be conducted to detect the three antibiotics with simultaneous qualitative and quantitative analyses in antigen-coated microtiter plate wells. The detection limit for each antibiotic was approximately 0.005 ng/mL, and the average recoveries of the multicolor QD were 88.10%–98.50%.

Taranova et al. [58] established an immunochromatographic test using multicolor QDs as the signal probe for the concurrent detection of several antibiotics, such as CAP, STM, ofloxacin (OFL), and fluoroquinolone chemicals (Fig. 4C) in milk. The basic design of the system was a “traffic light” format including three lines of different colors on a test strip. The mechanism utilized different monoclonal antibodies labeled with QDs and worked via the binding between antibiotics in the sample and the antibiotic-protein conjugates immobilized on the working membrane surface. Therefore, specific analytes could be identified by the specific color formed, and with increasing concentration of analytes in the sample corresponding to a gradual decrease in color intensity, concentrations of analytes could be determined by the fluorescence intensity of the lines. The LOD values for OFL, CAP, and STM were 0.3, 0.12, and 0.2 ng/mL, respectively, which were much lower than those of the regular immunoenzymatic assay.

### 3.4 Others

**Fig. 5:**
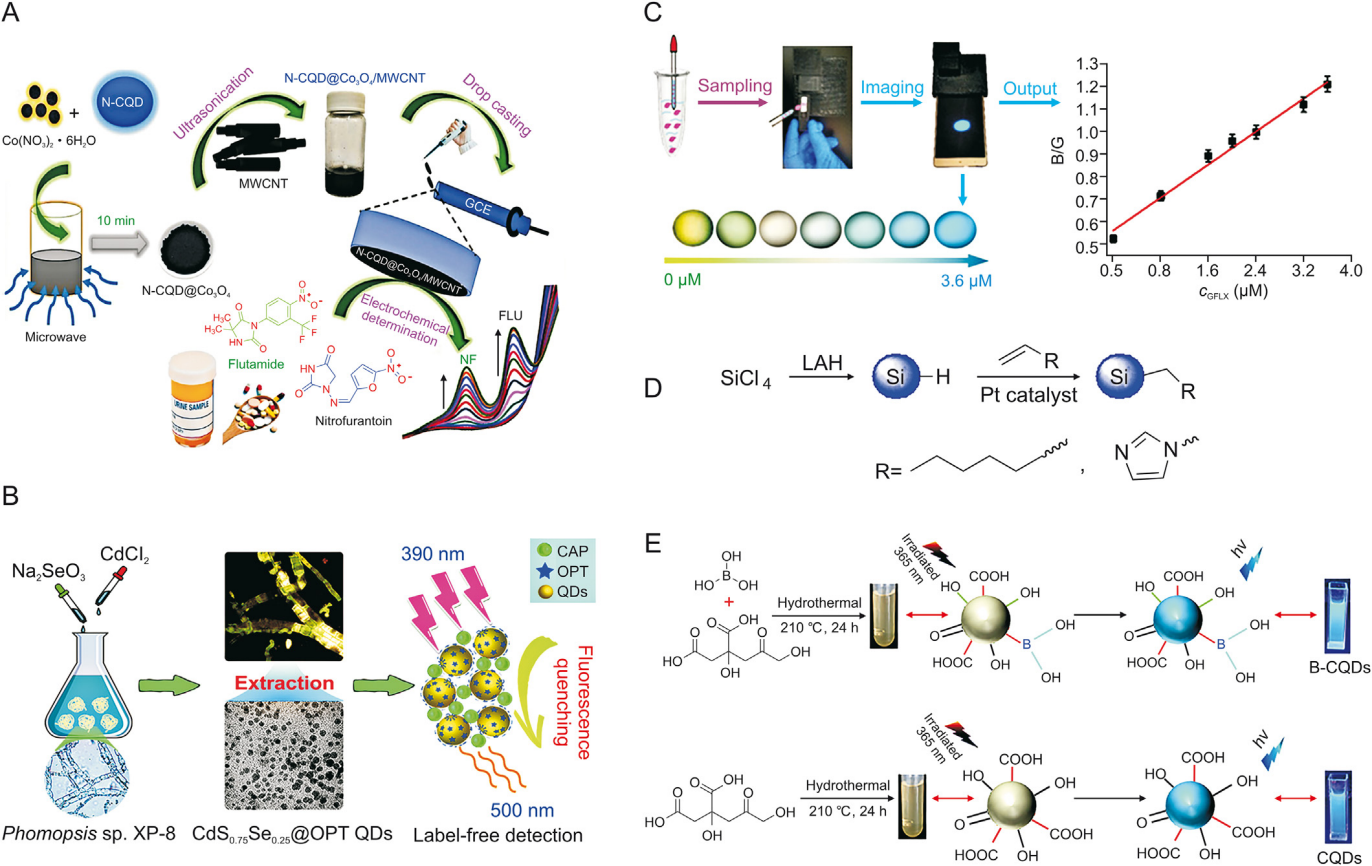
(A) Illustration for the synthesis of hybrid nanocomposite and its application for simultaneous electrochemical detection of flutamide and nitrofurantoin. (B) Schematic diagram of detection of chloramphenicol using CdS_*X*_Se_1−*X*_ QDs. (C) The operation process of the portable smartphone-based QD-coated filter paper for gatifloxacin (GFLX) sensing and output ratiometric fluorescent images at concentrations from 0 to 3.6 μM. (D) Synthesis routes of Si QDs capped with 1-heptene and 1-vinylimidazole. (E) Preparation schematic of the boron-doped carbon QDs (B-CQDs) and CQDs. (Reprinted from Refs. [[58], [59], [60],^68^,^78^] with permission).

Residual nitroaromatic compounds can cause serious health problems such as hematuria, prostatitis, drowsiness, nausea, methemoglobinemia, and liver dysfunction. Muthusankar et al. [58] developed an electrochemical sensor to simultaneously detect the anticancer drug flutamide (FLU) and the antibiotic nitrofurantoin (NF). The sensor used GCE embellished with the N-CQD-loaded Co_3_O_4_ (NCQD@Co_3_O_4_)/MWCNT blend nanocomposite (Fig. 5A) [58]. This nanocomposite was fabricated via a microwave synthesis method, with the N-CQD@Co_3_O_4_ hybridized with MWCNTs through an ultrasonication approach. Then, a steady N-CQD@Co_3_O_4_/MWCNT blend nanocomposite was posed with the hydrophobic interaction of the N-CQDs in the nanocomposite and MWCNTs. This hybrid nanocomposite was used as an electrode modifier for the simultaneous determination of FLU and NF, with a linear range of 0.05–590 μM and 0.05–1220 μM, and the LOD values were 0.0169 μM and 0.044 μM, respectively.

It has been reported that biosynthetic QDs using organisms provide a potential alternative for chemically synthesized QDs because of their simple and eco-friendly process. Xu et al. [59] designed a simple and green system to assemble CdS_x_Se_1−x_QDs biologically using *Phomopsis* sp. XP-8 (an endophytic filamentous fungus from *Eucommia ulmoides*) as a bioreactor for biosynthesizing nanocrystals (Fig. 5B). This endophyte has demonstrated the ability to synthesize gold nanoparticles, and could assemble CdS_*X*_Se_1−*X*_QD in an Na_2_SeO_3_ and CdCl_2_ composite aqueous solution. The resulting QDs were subsequently used as fluorescent nanoprobes to determine CAP with an LOD of 0.89 μg/L in a wide linear range of 3.13–500 μg/L. Owing to the aggregation of protein-coated QDs and CAP, the fluorescence quenching of CdS_0.75_Se_0.25_@OPT QDs by CAP was speculated to be a static quenching mechanism.

Ye et al. [60] developed a fluorescent sensing system utilizing smartphone-based ratiometric QDs for the on-site detection of gatifloxacin (GFLX) by transforming the fluorescent signal into visual RGB (i.e., red (R), green (G), and blue (B)) color display at a proper proportion (Fig. 5C). GFLX could launch the yellow-green fluorescence quenching of QDs via PET, and the built-in camera of the smartphone with a UV lamp could gather point-of-care images with mutative fluorescent color, which were further transformed to the numerical color RGB values based on the established Color Picker App. Smartphone fluorescence detection was successfully used for point-of-care and on-site monitoring of GFLX in food and environmental samples, with the advantages of short analysis time and good visualization. The LOD of GFLX by these proposed smartphone-based QDs was 0.26 nM, and the monitoring time was 5.0 min.

AMX is a semi-synthetic penicillin made from ampicillin with a moderate spectrum to treat certain infections caused by bacteria and is effective on an extensive range of gram-positive organisms and a limited range of gram-negative organisms [16,78]. Wang et al. [68] developed a system in which water-soluble Si QDs were covalently bonded with AMX to measure the efficiency of controlled-release drug delivery (Fig. 5D). AMX could strongly bond to the amine-Si QDs, as verified by the absorbance of 1650 cm^−1^ of the allylamine, and the prepared Si QDs were stable against oxidation in water with almost no detectable Si–O vibrational peak. These prepared Si QDs could act as effective drug delivery materials and offer the possibility of extended drug release; however, this Si QD delivery system lacked a complete evaluation of its pharmacokinetics, toxicity, and efficacy.

AMX is a popular antibiotic used to treat bacterial infections; however, this drug residue could pose a potential threat because of its misuse in bacterial inhibition, such as by causing drug resistance and even environmental pollution [78]. Hence, the ability to detect AMX and eliminate this drug has become a crucial problem. Doping can effectively adjust semiconductor energy levels, structures, surface states, optics, magnetism, and spintronics; doped QDs have better properties than the original QDs [79].

Using hydropyrolysis, Zhang et al. [78] fabricated B-CQDs as a probe for the detection of AMX, with citric acid as a carbon source and boric acid as a modifier (Fig. 5E). These QDs showed clear blue-fluorescence with a narrow average atom diameter of 2.3 nm and had multiple graphite phase structures including several functional groups, such as –OH, –COOH, –C=O, and −C−B bonds. B-CQDs acted as a fluorescence probe that achieved sensitive detection of AMX in an aqueous solution over a linear range of 1.43–429.12 μmol/L with an LOD of 0.825 μmol/L. In addition, this prepared probe displayed a remarkable selectivity, in which the fluorescence property was stable and was not interfered with by metal ions, saccharides, or amino acids.

## 4 Conclusion

In this review, we summarized the features of different types of QDs and their applications in antibiotics detection. First, the significance of antibiotics detection was described in detail, the variety of QDs used for the detection of antibiotics were briefly generalized and summarized, including comments, the limits of detection, and matrices, and the instances for the detection of antibiotics based on QDs were reviewed. Among the analytical methods based on QDs, the advantages of user-friendly operation, low cost, good sensitivity and selectivity, excellent stability, and fast response have been realized in antibiotics detection. To date, QDs have played significant roles in antibiotics detection, serving as internal references, energy donors, carrier vehicles, target-sensitive dyes, reference dyes, and recognition units in sensing systems. Next, QD-derived antibiotics sensors, including MIPs, aptamers, immunosensors, and other sensors, were described. MIPs possess the ability to recognize and bind specific target molecules. Aptamers have the advantages of high binding affinity, low immunogenicity, easy modification, and long-term stability. Immunosensors are characterized by unique selectivity, ease of use, flexible application, and great adaptability. These elements can enhance the ability of QD-based sensors to recognize target antibiotics, realizing the monitoring of antibiotics of interest in complex sample matrices.

As mentioned above, various antibiotics sensors based on QDs have displayed considerable advantages, including selectivity, sensitivity, simplicity, excellent visualization, and good portability. Despite the improvements achieved, there is still a need to prepare novel QDs with enhanced stability and optical properties. Additionally, the operation processes of the sensors are expected to be further simplified by the integration of portable optical devices. It is believed that by combining emerging sensing strategies, including CRISPR [39,96], MOF [97], and functional nucleic acids [98], QD-based antibiotics sensors will be applied as routine analytical tools in the future.

## CRediT author statement

**Rui Ding:** Writing - Original draft preparation, Reviewing and Editing, Visualization; **Yue Chen:** Writing - Reviewing and Editing, Validation, Supervision; **Qiusu Wang:** Investigation, Supervision; **Zhengzhang Wu:** Resources, Investigation; **Xing Zhang:** Conceptualization, Resources; **Bingzhi Li:** Conceptualization, Validation, Writing - Reviewing and Editing, Resources, Funding acquisition; **Lei Lin:** Conceptualization, Resources, Funding acquisition.

## Declaration of competing interest

The authors declare that there are no conflicts of interest.
